# Association between genetic polymorphisms and platinum-induced ototoxicity in children

**DOI:** 10.18632/oncotarget.25767

**Published:** 2018-07-20

**Authors:** Gabrielle Lui, Naïm Bouazza, Françoise Denoyelle, Marion Moine, Laurence Brugières, Pascal Chastagner, Nadège Corradini, Natacha Entz-Werle, Cécile Vérité, Judith Landmanparker, Hélène Sudour-Bonnange, Marlène Pasquet, Arnauld Verschuur, Cécile Faure-Conter, François Doz, Jean-Marc Tréluyer

**Affiliations:** ^1^ University of Paris Descartes, EA 7323, Sorbonne Paris-Cité, France; ^2^ CIC-1419 Inserm, Cochin-Necker, Paris, France; ^3^ Clinical Research Unit of Paris Descartes Necker Cochin, AP-HP, Paris, France; ^4^ Department of Pediatric Otolaryngology, Necker Children's Hospital, Paris, France; ^5^ Department of Children and Adolescents Oncology, Gustave Roussy, Villejuif, France; ^6^ Department of Pediatric Onco-Hematology, Children's Hospital, Vandoeuvre Les Nancy, France; ^7^ Pediatric Oncology Department, Mother-Children Hospital, Nantes, France; ^8^ Pediatric Onco-Hematology Unit, CHU of Strasbourg, Strasbourg, France; ^9^ Pediatric Hematology Department, Bordeaux University Hospital, Bordeaux, France; ^10^ Sorbonne University, Department of Pediatric Hematology Oncology, APHP, Trousseau Hospital, Paris, France; ^11^ Pediatric Oncology Unit, Children, Adolescents and Young Adults Unit, Oscar Lambret Center, Lille, France; ^12^ Children's Hospital, University Hospital of Toulouse, Toulouse, France; ^13^ Pediatric Oncology Department, La Timone Children's Hospital, Marseilles, France; ^14^ Institute of Pediatric Hemato-Oncology, Lyon, France; ^15^ Oncology Center SIREDO, Care, Innovation and Research for Children, Adolescents and Young Adults with Cancer, Curie Institute, Paris, France; ^16^ Paris Descartes University, Paris, France; ^17^ Department of Clinical Pharmacology, Cochin Hospital AP-HP, Paris, France

**Keywords:** platinum, pharmacogenetics, ototoxicity, children, cancer

## Abstract

Platinum is extensively used in the treatment of several childhood cancers. However, ototoxicity is one of the most notable adverse effects, especially in children. Several studies suggest that genetics may predict its occurrence. Here, polymorphisms associated with platinum-induced ototoxicity were selected from the literature and were investigated in a pediatric population treated with platinum-based agents. In this retrospective study, patients treated with cisplatin and/or carboplatin were screened. The patients with pre- and post-treatment audiogram (Brock criteria) available were included. We selected polymorphisms that have previously been associated with cisplatin ototoxicity with a minor allele frequency ≥30%. Deletion of *GSTM1* and *GSTT1*, rs1799735 (*GSTM3*), rs1695 (*GSTP1*), rs4880 (*SOD2*), rs2228001 (*XPC*), rs1799793 (*XPD*) and rs4788863 (*SLC16A5*) were investigated. Data of one hundred and six children matching the eligible criteria were analyzed. Thirty-three patients (31%) developed ototoxicity (with a Brock grade ≥2). The probability of hearing loss increased significantly in patients carrying the null genotype for *GSTT1* (P = 0.03), A/A genotype at rs1695 (P = 0.01), and C/C genotype at rs1799793 (P = 0.008). We also showed an association of the cumulative doses of carboplatin with cisplatin ototoxicity (P <0.05).

To conclude, deletion of *GSTT1*, rs1695 and rs1799793 may constitute potential predictors of platinum-induced ototoxicity.

## INTRODUCTION

Platinum is an essential component of chemotherapies in a wide range of pediatric cancers, such as osteosarcoma, neuroblastoma, medulloblastoma, germ cell tumors, retinoblastoma or hepatoblastoma [1]. Its use has been approved despite several side effects, among which ototoxicity is one of the most relevant. After a same dose of platinum some patients may suffer from long-lasting hearing loss whereas others may not. Risk factors are not well determined. Several clinical factors have been shown to increase the susceptibility to cisplatin-induced ototoxicity. Males may be more prone to develop ototoxicity than females [2–4]. Futhermore, younger children may be more sensitive than adults [5], and may suffer from speech and social development impairment. Combined radiotherapy and cumulative doses of cisplatin have also been identified as risk factors [3, 5, 6]. But these criteria are insufficient to identify with accuracy patients with high hearing loss risk. It is therefore necessary to better determine the predisposing factors in order to protect children from cisplatin related deafness.

Growing number of studies suggest that genetics may be a relevant factor in ototoxicity, but results are contradictory and scarce for children [7]. One of the cisplatin cytotoxic mechanisms is to induce oxidant stress generating reactive oxygen species [8], from which cochlea cells are protected by a high expression of antioxidant enzymes, like glutathione-S-transferases (GST), or superoxide dismutases (SOD). A deletion of 3 nucleotides on *GSTM3* gene [9] has been shown to have a protective role, whereas having *GSTT1* and *GSTM1* genes and the A/A genotype at rs1695 in *GSTP1* has been associated with hearing loss [7, 10]. In other studies, the presence of *GSTT1* [11], and the AG or GG genotypes at rs1695 have been correlated with a greater risk of severe hearing impairment [12]. The manganese SOD, encoded by the *SOD2* gene, catalyzes the conversion of O2-. to H2O2 and O2. [8]. A study in medulloblastoma correlated the SNP rs4880 in *SOD2* with ototoxicity in adults [13].

Another cytotoxic mechanism of platinum components is to form adducts in DNA, inducing apoptosis and, in the same time, activating nucleotide excision repair (NER)[14, 15]. NER envolves several xeroderma pigmentosum (XP) complementing proteins. The SNPs rs1799793 and rs2228001, in *XPD* and *XPC* genes respectively, have been correlated with cisplatin induced hearing loss [16, 17].

Cisplatin and carboplatin are transported through different influx transporters coded by the *solute carrier* (*SLC)* family genes [18–20]. Several works associated *SLC* genes and ototoxicity. The T allele at rs316019 in *SLC22A2* has been shown to protect from hearing loss [21]. The C allele carriers at rs10981694 in *SLC31A1* had an increased susceptibility to ototoxicity [22]. The rs4788863 in *SLC16A5* has also been correlated with hearing loss in adults [23]. The A allele at the rs2075252 in megaline gene had been associated with hearing impairment by Riederman but not replicated by Choeyprasert [11, 24].

Other genes with a lesser-understood biologic role in ototoxicity have been identified. Ross’ work has associated several SNPs in *thiopurine S-methyltransferase* (*TPMT*) and *catechol O-methyltransferase* (*COMT*) [25, 26] with ototoxicity. However, these results were not confirmed by Yang et al. [4]. *ACYP2* gene has been associated with hearing loss in a GWAS study [27], and replicated in another gene candidate study [28].

Here, we aimed to investigate the most common polymorphisms with a minor allele frequency (MAF) ≥30% selected from the literature, in a pediatric population treated with platinum without cranial irradiation.

## RESULTS

### Population characteristics

**Figure 1:**
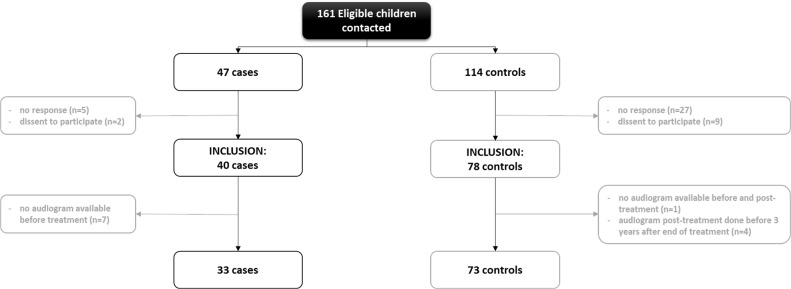
Flow chart of patient selection and inclusion to the study In grey, number of patients excluded and the reasons.

A total of 161 eligible patients/families were contacted for the study. A signed informed consent was provided by 43 adults and 75 parents (or guardians) for their child. However, 12 children did not satisfy the inclusion/exclusion criteria and were filtered out (Figure 1). At the end, 106 patients were included in the analysis.

Patients’ characteristics are presented in Table 1. Thirty-three patients (31%) displayed a moderate or severe hearing loss (Brock grade≥ 2) after treatment. The time elapsed between end of treatment and last audiogram test is given in Table 1 (Median (IQR) for cases: 6 [2–8] and for controls: 6 [4–9] years). The medians of age and of bodyweight at the beginning of the treatment was 2.5 years (range: 0.2-16.9) and 14.4 kg (range: 4.5-100), respectively. Sixty patients (56.6%) received cisplatin, 10 (9.4%) received carboplatin, and 36 (34%) received both cisplatin and carboplatin. The median cumulative dose of cisplatin was 400 mg/m^2^ (range: 44-650) and 1518 mg/m^2^ (range: 278-5040) for carboplatin. Children were treated for osteosarcoma (11%), retinoblastoma (8%), hepatoblastoma (18%), neuroblastoma (27%) or malignant germ cell tumor (35%). No difference regarding age, bodyweight or sex was evidenced between the patients who developed ototoxicity and the other patients. However, the distribution of the type of tumors (P <10^−4^) and the percentage of patients treated with carboplatin (P =0.03) were different between patients with hearing loss and control patients.

**Table 1:** Characteristics of the patients

	Brock grade ≥2 n=33	Brock grade =0 n=73	P-value
**Sex**			
Female	14 (42.4%)	43 (58.9%)	0.17
**Age class at treatment initiation**			0.74
0-23 months	13 (39.4%)	25 (34.2%)	
2-5 years	10 (30.3%)	18 (24.7%)	
6-12 years	4 (12.1%)	10 (13.7%)	
>12 years	6 (18.2%)	20 (27.4%)	
**Age at treatment initiation (years)**	2.2 [1.8-10.4]	2.7 [1.2-12.2]	0.72
**Bodyweight at treatment initiation (kg)**	12.9 [11.3–28]	14.5 [10.4-47]	0.54
**Primary tumor**			<10^−4^
Neuroblastoma	15 (45.5%)	14 (19.2%)	
Hepatoblastoma	2 (6.1%)	17 (23.3%)	
Retinoblastoma	3 (9.1%)	6 (8.2%)	
Malignant germinal tumor	5 (15.2%)	32 (43.8%)	
Osteosarcoma	8 (24.2%)	4 (5.5%)	
**Treatments**			
Cisplatin	30 (90.9%)	66 (90.4%)	1
Cisplatin cumulative dose (mg/m^2^)	400 [330-426.8]	353 [300–480]	0.39
Carboplatin	20 (60.6%)	26 (35.6%)	0.03
Carboplatin cumulative dose (mg/m^2^)	1550 [1382.5-2175]	1518 [1261.5-2500]	0.82
Ototoxic antibiotics (aminoside, glycopeptide)	29 (100%)	46 (97.9%)	1
Ototoxic diuretics (furosemide)	3 (10.3%)	10 (21.3%)	0.35
**Time elapsed between end of treatment and last audiogram test (years)**	6 [2–8]	6 [4–9]	0.21
**Brock classification**			-
Grade 0	0 (0%)	73 (100%)	
Grade 1	0 (0%)	0 (0%)	
Grade 2	11 (33.3%)	0 (0%)	
Grade 3	18 (54.5%)	0 (0%)	
Grade 4	4 (12.1%)	0 (0%)	

key : Median [IQR] for quantitative data and number (percentage) for categorical data.

### Genetic analysis

All SNPs passed the quality control checks (i.e. Hardy-Weinberg equilibrium with P > 0.05, MAF ≥30% and percentage of missing genotype <5%, see Table 2). The repartition of genotypes in controls and cases was represented in Table 3.

**Table 2:** SNPs genotyping quality and control checks

Gene	SNPs	chr.	Alleles Maj/Min	% Missing	Observed MAF	HWE^*^ (P-value)
**GSTP1**	rs1695	11	A/G	0	0.32	0.50
**SOD2**	rs4880	6	A/G	0.9	0.49	0.33
**XPC**	rs2228001	3	T/G	0	0.43	0.43
**ERCC2**	rs1799793	19	C/T	0	0.33	1.0
**SLC16A5**	rs4788863	17	C/T	0	0.29	1.0

^*^Hardy Weinberg Equilibrium.

**Table 3:** Association between Brock grade and genetic polymorphisms in the univariate analysis

	Brock grade ≥2 n=33	Brock grade =0 n=73	P-value
**GSTM1**			0.27
null	18 (54.5%)	29 (40.8%)	
**GSTT1**			0.08
null	11 (33.3%)	11 (15.9%)	
**rs1799735 (GSTM3)^1^** (number of deletions)			0.31
0	19 (57.6%)	51 (69.9%)	
1	10 (30.3%)	18 (24.7%)	
2	4 (12.1%)	4 (5.5%)	
**rs1695 (GSTP1)**			0.046
AA	21 (63.6%)	29 (39.7%)	
AG	8 (24.2%)	35 (47.9%)	
GG	4 (12.1%)	9 (12.3%)	
**rs4880 (SOD2)**			0.48
AA	10 (31.2%)	20 (27.4%)	
AG	16 (50%)	31 (42.5%)	
GG	6 (18.8%)	22 (30.1%)	
**rs2228001 (XPC)**			0.17
GG	4 (12.1%)	18 (24.7%)	
GT	19 (57.6%)	29 (39.7%)	
TT	10 (30.3%)	26 (35.6%)	
**rs1799793 (ERCC2)**			0.05
CC	20 (60.6%)	27 (37%)	
CT	9 (27.3%)	38 (52.1%)	
TT	4 (12.1%)	8 (11%)	
**rs4788863 (SLC16A5)**			0.22
CC	21 (63.6%)	33 (45.2%)	
CT	10 (30.3%)	33 (45.2%)	
TT	2 (6.1%)	7 (9.6%)	

^1^ P=0.25 for 0-1 versus 2 deletions and P=0.31 for 0 versus 1-2 deletions.

**Figure 2:**
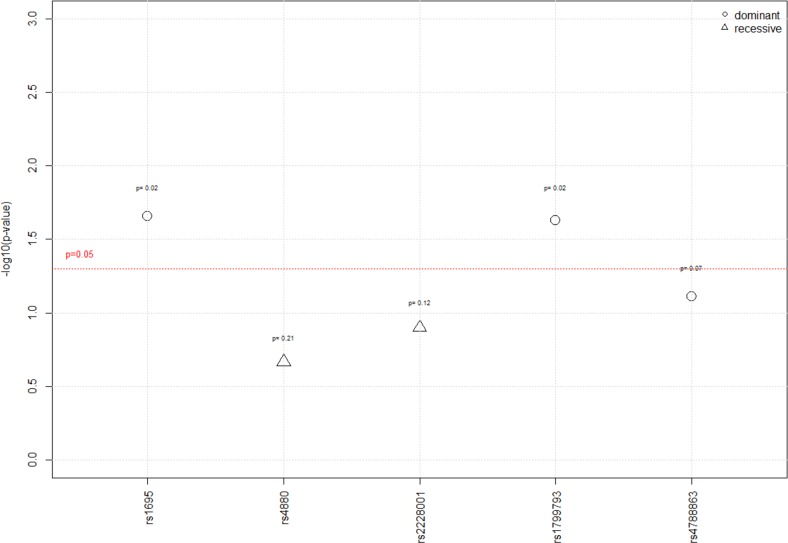
Results of the univariate analysis according to the 3 genetic models For each SNP, the p-value reported was the most significant between the three genetic models (additive, recessive, and dominant models).

They were further tested in a univariate analysis for association with hearing loss, following different inheritance models (additive, dominant and recessive) (Figure 2).

Under a dominant genetic model, the percentage of patients with a grade ≥2 on the Brock scale was significantly higher in the group carrying the homozygote wild type A/A genotype (rs1695) in *GSTP1* than in the group carrying A/G or G/G genotypes, i.e. 64% vs. 40% (P=0.02). The rs1799793 in *XPD/ERCC2* gene was also significantly associated with hearing loss under a dominant model. A significantly higher percentage of homozygote C/C wild type was observed in children who experienced hearing loss after chemotherapy when compared to the patients carrying C/T or T/T genotypes (61% versus 37%; P=0.02). Furthermore, a trend regarding the association between *GSTT1* null genotype and hearing loss was also observed in the univariate analysis. The *GSTT1* deletion was more frequent in the group of patients with a grade ≥2 (33% vs. 16%; P=0.08), but was not significant. As well, a statistical trend between rs4788863 in *SLC16A5* gene and hearing loss under a dominant genetic model hypothesis was shown (P=0.07). No association was found with any of the other polymorphisms on *GSTM1, GSTM3, SOD2* and *XPC*.

According to these results, the following polymorphisms were further tested under a dominant genetic model in a multivariate analysis: *GSTT1* deletion, rs1695, rs1799793 and rs4788863 (Table 4). The multivariate logistic regression confirmed the significant association between *GSTT1* deletion, *GSTP1* (rs1695), and *ERCC2* (rs1799793) genes and hearing loss after chemotherapy. The patients with *GSTT1* gene deletion had a higher risk to develop ototoxicity (OR 3.53; 95% CI 1.07–11.58; P = 0.03). Patients who were A/A wild type - *GSTP* had also a higher risk of hearing loss (OR 3.76; 95% CI 1.33–10.61; P = 0.01). Furthermore, the patients with the C/C wild type genotype at rs1799793 in *ERCC2* had significantly 4.1 times higher risk to develop ototoxicity (95% CI 1.43–11.52; P = 0.008). No significant interaction between these 3 polymorphisms was evidenced (P>0.05).

**Table 4:** Factors associated with Brock grade ≥2 in the multivariate analysis

	OR	IC 95%	P-value
GSTT1 (null genotype)	3.53	1.07 - 11.58	0.038
rs1695 (A/A vs. A/G or G/G)	3.76	1.33 - 10.61	0.012
rs1799793 (C/C vs. C/T or TT)	4.07	1.43 - 11.52	0.008
rs4788863 (C/C vs. C/T or TT)	2.16	0.79 - 5.93	0.136
Age at treatment initiation (years)	1.02	0.93 - 1.13	0.679
Carboplatin cumulative dose	1.06^*^	1 - 1.12	0.033
Cisplatin cumulative dose	1.68^*^	1.12 - 2.52	0.012

^*^OR expressed for each 100-unit dose increase.

As expected, the cumulative doses of carboplatin and cisplatin were also associated with grade ≥2, P=0.033 and P=0.012 respectively. For each 100 mg/m^2^ drug dose increase, the risk was increased by 1.68 for cisplatin and of 1.06 for carboplatin. In our study, the cumulative doses for cisplatin and carboplatin ranged from 44 to 650 mg/m^2^ and from 278 to 5040 mg/m^2^ respectively. For patients receiving both drugs, the median (range) for carboplatin dose was 1500 (278 – 2500) mg/m^2^. The median (range) for cisplatin dose was 395 (44 – 600).

## DISCUSSION

In this retrospective study, we investigated 8 polymorphisms in a pediatric population treated with platinum chemotherapy. We showed that 3 of them, rs1695 in *GSTP1*, the absence of *GSTT1*, and rs1799793 in *XPD/ERCC2* were significantly associated with hearing impairment, whereas rs4880, rs2228001, rs4788863, rs1799735, and presence of *GSTM1* were not.

GST family isoenzymes eliminate free radicals [8, 29], and increase cell resistance against platinum. They conjugate glutathione with xenobiotics, thus sequestrate platinum in the cytoplasm and prevent drug from entering into the nucleus to form DNA adducts [30, 31]. In our study, the presence of *GSTT1* was significantly associated with hearing protection, which is in accordance with its physiologic role, but not with previous studies [9–11]. An analysis on a small group of children with solid tumor showed a correlation between ototoxicity and presence of *GSTT1*, however no correlation for *GSTM1* was demonstrated [11]. In our study, the A/A genotype at rs1695 in *GSTP1* was also associated with hearing impairment, which is in agreement with Oldenburg et al [10]. Patients with A/G or G/G genotypes, ^105^Ile/^105^Val or ^105^Val/^105^Val on the amino acid sequence respectively, were less likely to develop ototoxicity suggesting a protective role of the Val variant. These findings were in accordance with Ishimoto et al. who reported an increased cytoprotection of the Val variant against cisplatin *in vitro* [32].

The mechanisms leading to inner ear cell apoptosis are not completely understood but may not be different from those induced in tumors. In addition to oxidative stress generation, cisplatin form adducts on DNA in the cochlear cells, leading to DNA repair enzymes activation and apoptosis [14], as shown in mice model. These enzymes play a central role in tumor resistance and may, as well, protect ear cells from apoptosis. In human, proteins implicated in NER system have been shown to protect from cisplatin induced apoptosis [15]. DNA repair involves several proteins which may retrieve DNA adducts [33], like Xeroderma Pigmentosum (XP) complementing proteins or differently named Excision Repair Cross-Complementing (ERCC) proteins. Extensively examined in different types of cancer, ERCC proteins have consistently been correlated with response to cisplatin therapy [17, 34]. Many polymorphisms have been correlated with survival, or response [17, 35, 36]. We investigated two polymorphisms on *ERCC1* and *ERCC2* previously found to be correlated with ototoxicity [16, 17]. Only rs1799793 on *ERCC2* was correlated with ototoxicity: the presence of two G alleles was associated with hearing loss. It is located in an exon, inducing an exchange of an aspartic acid to an asparagine amino acid, and has been correlated with increased cancer risk [37]. Our result is in line with several clinical studies, suggesting that homozygote GG at rs1799793 may respond better to platinum treatment, suggesting higher susceptibility of cells to cisplatin [38, 39]. This is in contradiction with the prior results of Lopes’ study [16], where they found an association of the TT genotype with moderated and severe deafness. In this latter study, classification of hearing loss (CTCAE) and analyzed population differed, including adults with head and neck irradiation.

Peters et al. [9] showed an association between GSTM3^*^B (rs1799735) with protection from cisplatin ototoxicity. However, in our study, no association was found, probably due to the difference in the hearing loss scale used to classify the patients and the size of the population. Due to the small sample size of patients enrolled in their study (39 patients), no multivariate analysis was performed to confirm this association.

We also investigated the rs4880 located in *SOD2*, previously described as being associated with ototoxicity by Brown et al. [13], but failed to confirm these previous results. Their study was done on pediatric patients treated for medulloblastoma, who also received radiotherapy. Radiotherapy may induce different chemical species. More superoxide anions may be generated, involving preferentially other elimination pathways, thereby different enzymes and polymorphisms. This enzyme may play a minor role in the detoxification of cisplatin without radiation, as patients who had received radiations were excluded from our study.

Cisplatin is transported into the inner ear cells through different transporters. Considering the size of our population, only the most frequent polymorphism, rs478886 on *SLC16A5* gene (coding for the monocarboxylate transporter 6) was investigated. This SNP has been highlighted in an adult population of men treated for germ cell testicular cancer (OR= 0.05) [23]. In our study, this SNP was not significantly associated to ototoxicity. This suggests that, in children, mechanisms may be different and the impact of this mutation weaker compared to adults. Adults and children express enzymes and transporters at different level therefore are differently sensitive to chemotherapies developing specific side effects. In the same way, adults are more prone to develop cisplatin related peripheral neurotoxicity than younger patients [40].

One limit of this study is the number of patients - higher than the several prior studies- but smaller with respect to Yang's or Pussegoda's ones [4, 26]. This limited the number of polymorphisms studied. To have enough statistic power, we have chosen a MAF threshold of 30%, hence excluding several SNPs previously associated with cisplatin induced hearing loss. We did not include the SNPs in the following genes: *ACYP2* (coding for an acylphosphatase) [41], *SLC31A1* gene coding for the copper transport protein 1 [22], *SLC22A2* coding for the organic cation transport protein 2 [21], *megalin* [24], *TPMT* and *COMT* [25, 26, 41] and *Mendelian deafness* gene [42].

The proportion of cases in our study is 31%. Our results are consistent with Yancey's and Olgun's studies which reported 28% and 30% of children with Brock grade ≥2 respectively [2, 3]. These results may be different from other studies, mainly due to the method used to grade the hearing loss.

In our cohort, tumor type distribution is different between cases and controls. But it does not seem likely that it impacts our results since ototoxicity is due to the treatment itself. Radiation used to treat certain types of tumor, which could have been a confounding factor, was excluded. Furthermore, it is noteworthy that genotypes for all selected polymorphisms were well-balanced across the different type of tumors (*P* > 0.05).

As expected and previously reported, we also showed an association between ototoxicity and cumulative doses of cisplatin [3, 5, 6] and, to a lesser extent, of carboplatin. The latter was suggested to be associated with a much lower risk of ototoxicity than cisplatin [43].

Nowadays, more children are successfully treated for cancer, but it is still difficult to protect them from cisplatin-induced deafness which compromises their development and quality of life. Our work suggests that three polymorphisms in *GSTT1*, *GSTP1* and *ERCC2* may constitute potential biomarkers of platinum induced ototoxicity. But fusion of existing data bases devoted to platinum's ototoxicity as well as larger prospective clinical trials have to be conducted to confirm the potential predictive value of these polymorphisms. They might be then used to discriminate high-risk patients, to whom could be proposed new preventing strategies [44].

## MATERIALS AND METHODS

### Patients, treatment and clinical variables

The Otoplat protocol was approved by the Ethics Committee of Ile de France III (ClinicalTrials.gov. Identifier: NCT02425397). In this retrospective study, which started in March 2011 and ended in January 2016, we examined medical records of children treated with cisplatin and/or carboplatin in 10 French pediatric cancer departments: Curie Institute (Paris), University Hospital of Nancy, University Hospital of Nantes, University Hospital of Strasbourg, Institute of Pediatric Hemato-Oncology (Lyon), Gustave Roussy Institute (Villejuif), Trousseau Hospital (Paris), Oscar Lambret Center (Lille), Children Hospital (Toulouse) and Timone Hospital (Marseilles).

We screened patients with pure tone audiometry performed in a sound-proof room, using visual reinforcement audiometry in youngest children. We selected patients with audiogram tests done before treatment and at least 3 years after the end of the treatment. Exclusion criteria were defined as follows: patients i) with cerebral tumors, ii) with parameningeal rhabdomyosarcoma, iii) with renal toxicity (grade ≥ 2) during treatment (Common Terminology Criteria for Adverse Events), iv) with a pathological audiogram before treatment, v) who have undergone facial, cerebral or total body irradiation, vi) with Brock grade 1 to better discriminate the severe hearing loss from no impairment.

An information note along with an informed consent was sent to all eligible patients/parents. Patients (or parents who agreed to their child's participation in the study) were asked to send back the swab used to collect mucosa cells along with the signed informed consent form.

Hearing loss was assessed using the Brock criteria [45], a classification specifically designed for cisplatin-related ototoxicity. The results obtained from the better ear or the free field tests were used to define the grade. Hearing loss was graded from 0 to 4. Patients were classified in the group platinum-related ototoxicity if they had a grade 2, 3 or 4 on the Brock scale after the end of treatment, and in the control group, those with a grade 0, with no hearing impairment.

### DNA extraction and genotyping

DNAs were extracted from swabs following manufacturer's instructions, using Gentra Puregene Buccal cell kit (Qiagen). To sum up the procedure, swabs were incubated 1 hour in cell lysing solution at 65°C then after addition of K proteinase, at 55°C during 2.5 hours. DNAs were precipitated with ethanol, centrifuged, washed in 70% isopropanol twice and then dissolved in hydration solution. DNAs were quantified by spectrophotometer (Nanodrop, ThermoScientific).

In this association study with candidate genes, the polymorphisms were selected based on literature review. Thereafter, SNPs with a minor allele frequency lower than 30% were filtered out. This MAF value would guaranty an 80% power to detect at least an Odds Ratio of 2.3, 3.6 or 4.7 using an additive, dominant or recessive genetic model respectively.

GSTM3 rs1799735 (a deletion of 3 base pairs) was determined by PCR using a predesigned LightSNiP assay (Tib MolBio, Germany) in LightCycler 480 Probes Master Mix (Roche), according to manufacturer's instruction on a LightCycler 480 System (Roche) using 40 ng of DNA. The PCR conditions consisted of an initial denaturation step of temperature of 95°C (10 minutes), followed by 45 cycles of melting (10 seconds at 95°C), annealing (10 seconds at 60°C), and extension (15 seconds at 72°C). After the PCR, a melting curve between 40°C and 95°C was realized, to assess the melting temperatures (Tm) of amplicons. Tms were observed at 48°C and 61°C, corresponding, respectively, to the deletion and the normal genotype.

GSTM1 and GSTT1 were simultaneously amplified from 50ng of DNA in a multiplex PCR in SYBER^®^ green on a LightCycler 480 System (Roche), following a method modified from Barahmani's work [46], using specific primers : forward 5′-GAACTCCCTGAAAAGCTAAAGC-3′, reverse 5′-GTTGGGCTCAAATATACGGTGG-3′ for *GSTM1* at 0.5μM, forward 5′-TTCCTTACTGGTCCTCACATCTC-3′, reverse 5′-TCACCGGATCATGGCCAGCA-3′, for *GSTT1* at 0.3μM, and as control *Bcl2*, forward 5′-GCAATTCCGCATTTAATTCATGG-3′, reverse 5′-GAA-ACAGGCCACGTAAAGCAAC-3′ at 0.5μM. The PCR conditions consisted of an initial melting temperature of 95°C (10 minutes), followed by 33 cycles of melting (10 seconds at 95°C), annealing (30 seconds at 62°C), and extension (25 seconds at 72°C). A melting curve between 65°C and 95°C was realized to assess the Tm of the amplification products (Bcl2 Tm at 78°C, GSTM1 Tm at 82°C and GSST1 Tm at 87°C). Bcl2 Tm was a positive control of PCR for each patient.

The other SNPs rs1695 (*GSTP1*), rs4880 (*SOD2*), rs2228001 (*XPC*), rs1799793 (*ERCC2*), rs4788863 (*SLC16A5*) were genotyped using predesigned TaqMan assay (Life technologies) following manufacture's instructions. q-PCR were performed in 25 μl on 35.5 ng of DNA using TaqMan^®^ Universal Master Mix II, with UNG, on an Applied biosystem 7500 Real Time PCR system (Applied Biosystems). The thermal cycling comprised a holding stage (10 minutes at 95°C) and 45 cycles of denaturation (15 seconds at 92°C) and annealing/extension (1 minute at 60°C). Analysis was performed on the Sequence Detector Software (SDSv2.0, Applied Biosystems). Genotypes were determined by an endpoint fluorescence reading.

### Statistical analysis

Statistical analysis was performed with *ad hoc* routines implemented in R software (http://www.R-project.org). The data are presented as proportions for categorical data and as median, interquartile range (IQR) and range for quantitative data. The primary endpoint was defined as moderate or severe hearing loss after the end of treatment (with a grade ≥2 on the Brock scale).

Quantitative variables were compared with the non-parametric Wilcoxon tests and proportions with the Fisher's exact tests or the chi-squared tests, as appropriate. Logistic regression models were used to test all possible genetic inheritance models (i.e., additive, dominant, and recessive) for all selected polymorphisms. *GSTM1*, *GSTM3* and *GSTT1* polymorphisms were analyzed as categorical covariate to evaluate the association between these gene deletions and hearing loss. A multivariate logistic regression was then used. All SNPs with a P value < 0.10 in the univariate analysis were included in the multivariate model.

Both cumulative doses of cisplatin and carboplatin were used as continuous covariate in the multivariate logistic regression model. For patients treated with one drug, the unused drug dose was set to zero. For patients receiving both drugs, the two cumulative doses were used.

The multivariate analysis was adjusted for known confounding variables (i.e., age at diagnosis, cisplatin cumulative dose and carboplatin cumulative dose). Associations were expressed by ORs with their respective 95% confidence interval (95% CI). All statistical tests were two sided, and P < 0.05 was defined as statistical significance.

## Abbreviations

CI: confidence interval
CTCAE: common terminology criteria for adverse events
GST: glutathione-S-transferase family
ERCC: excision repair cross-complementary genes
MAF: minor allele frequency
OCT: organic cation transport protein
OR: odds ratio
SOD: superoxide dismutase
SLC: solute carrier
SNPs: single nucleotide polymorphisms
XP: xeroderma pigmentosum

## References

[1] Ho GY, Woodward N, Coward JIG (2016). Cisplatin versus carboplatin: comparative review of therapeutic management in solid malignancies. Crit Rev Oncol Hematol.

[2] Olgun Y, Aktaş S, Altun Z, Kırkım G, Kızmazoğlu DÇ, Erçetin AP, Demir B, İnce D, Mutafoğlu K, Demirağ B, Ellidokuz H, Olgun N, Güneri EA (2016). Analysis of genetic and non genetic risk factors for cisplatin ototoxicity in pediatric patients. Int J Pediatr Otorhinolaryngol.

[3] Yancey A, Harris MS, Egbelakin A, Gilbert J, Pisoni DB, Renbarger J (2012). Risk factors for cisplatin-associated ototoxicity in pediatric oncology patients. Pediatr Blood Cancer.

[4] Yang JJ, Lim JY, Huang J, Bass J, Wu J, Wang C, Fang J, Stewart E, Harstead EH, Shuyu E, Robinson GW, Evans WE, Pappo A (2013). The role of inherited TPMT and COMT genetic variation in cisplatin-induced ototoxicity in children with cancer. Clin Pharmacol Ther.

[5] Li Y, Womer RB, Silber JH (2004). Predicting cisplatin ototoxicity in children: the influence of age and the cumulative dose. Eur J Cancer.

[6] Lanvers-Kaminsky C, Krefeld B, Dinnesen AG, Deuster D, Seifert E, Würthwein G, Jaehde U, Pieck AC, Boos J (2006). Continuous or repeated prolonged cisplatin infusions in children: a prospective study on ototoxicity, platinum concentrations, and standard serum parameters. Pediatr Blood Cancer.

[7] Langer T, am Zehnhoff-Dinnesen A, Radtke S, Meitert J, Zolk O (2013). Understanding platinum-induced ototoxicity. Trends Pharmacol Sci.

[8] Sheth S, Mukherjea D, Rybak LP, Ramkumar V (2017). Mechanisms of cisplatin-induced ototoxicity and otoprotection. Front Cell Neurosci.

[9] Peters U, Preisler-Adams S, Hebeisen A, Hahn M, Seifert E, Lanvers C, Heinecke A, Horst J, Jürgens H, Lamprecht-Dinnesen A (2000). Glutathione S-transferase genetic polymorphisms and individual sensitivity to the ototoxic effect of cisplatin. Anticancer Drugs.

[10] Oldenburg J, Kraggerud SM, Cvancarova M, Lothe RA, Fossa SD (2007). Cisplatin-induced long-term hearing impairment is associated with specific glutathione s-transferase genotypes in testicular cancer survivors. J Clin Oncol.

[11] Choeyprasert W, Sawangpanich R, Lertsukprasert K, Udomsubpayakul U, Songdej D, Unurathapan U, Pakakasama S, Hongeng S (2013). Cisplatin-induced ototoxicity in pediatric solid tumors: the role of glutathione S-transferases and megalin genetic polymorphisms. J Pediatr Hematol Oncol.

[12] Rednam S, Scheurer ME, Adesina A, Lau CC, Okcu MF (2013). Glutathione S-transferase P1 single nucleotide polymorphism predicts permanent ototoxicity in children with medulloblastoma. Pediatr Blood Cancer.

[13] Brown AL, Lupo PJ, Okcu MF, Lau CC, Rednam S, Scheurer ME (2015). SOD2 genetic variant associated with treatment-related ototoxicity in cisplatin-treated pediatric medulloblastoma. Cancer Med.

[14] Guthrie OW (2009). DNA repair proteins and telomerase reverse transcriptase in the cochlear lateral wall of cisplatin-treated rats. J Chemother.

[15] Lomonaco SL, Xu XS, Wang G (2009). The role of Bcl-x(L) protein in nucleotide excision repair-facilitated cell protection against cisplatin-induced apoptosis. DNA Cell Biol.

[16] Lopes-Aguiar L, Costa EF, Nogueira GA, Lima TR, Visacri MB, Pincinato EC, Calonga L, Mariano FV, de Almeida Milani Altemani AM, Altemani JM, Coutinho-Camillo CM, Ribeiro Alves MA, Moriel P (2017). XPD c.934G>A polymorphism of nucleotide excision repair pathway in outcome of head and neck squamous cell carcinoma patients treated with cisplatin chemoradiation. Oncotarget.

[17] Caronia D, Patiño-García A, Milne RL, Zalacain-Díez M, Pita G, Alonso MR, Moreno LT, Sierrasesumaga-Ariznabarreta L, Benítez J, González-Neira A (2009). Common variations in ERCC2 are associated with response to cisplatin chemotherapy and clinical outcome in osteosarcoma patients. Pharmacogenomics J.

[18] Ciarimboli G, Deuster D, Knief A, Sperling M, Holtkamp M, Edemir B, Pavenstädt H, Lanvers-Kaminsky C, am Zehnhoff-Dinnesen A, Schinkel AH, Koepsell H, Jürgens H, Schlatter E (2010). Organic cation transporter 2 mediates cisplatin-induced oto- and nephrotoxicity and is a target for protective interventions. Am J Pathol.

[19] Holzer AK, Katano K, Klomp LW, Howell SB (2004). Cisplatin rapidly down-regulates its own influx transporter hCTR1 in cultured human ovarian carcinoma cells. Clin Cancer Res.

[20] Ishida S, Lee J, Thiele DJ, Herskowitz I (2002). Uptake of the anticancer drug cisplatin mediated by the copper transporter Ctr1 in yeast and mammals. Proc Natl Acad Sci U S A.

[21] Lanvers-Kaminsky C, Sprowl JA, Malath I, Deuster D, Eveslage M, Schlatter E, Mathijssen RH, Boos J, Jürgens H, Am Zehnhoff-Dinnesen AG, Sparreboom A, Ciarimboli G (2015). Human OCT2 variant c.808G>T confers protection effect against cisplatin-induced ototoxicity. Pharmacogenomics.

[22] Xu X, Ren H, Zhou B, Zhao Y, Yuan R, Ma R, Zhou H, Liu Z (2012). Prediction of copper transport protein 1 (CTR1) genotype on severe cisplatin induced toxicity in non-small cell lung cancer (NSCLC) patients. Lung Cancer.

[23] Drögemöller BI, Monzon JG, Bhavsar AP, Borrie AE, Brooks B, Wright GEB, Liu G, Renouf DJ, Kollmannsberger CK, Bedard PL, Aminkeng F, Amstutz U, Hildebrand CA (2017). Association Between SLC16A5 Genetic Variation and Cisplatin-Induced Ototoxic Effects in Adult Patients With Testicular Cancer. JAMA Oncol.

[24] Riedemann L, Lanvers C, Deuster D, Peters U, Boos J, Jürgens H, am Zehnhoff-Dinnesen A (2008). Megalin genetic polymorphisms and individual sensitivity to the ototoxic effect of cisplatin. Pharmacogenomics J.

[25] Ross CJ, Katzov-Eckert H, Dubé MP, Brooks B, Rassekh SR, Barhdadi A, Feroz-Zada Y, Visscher H, Brown AM, Rieder MJ, Rogers PC, Phillips MS, Carleton BC (2009). Genetic variants in TPMT and COMT are associated with hearing loss in children receiving cisplatin chemotherapy. Nat Genet.

[26] Pussegoda K, Ross CJ, Visscher H, Yazdanpanah M, Brooks B, Rassekh SR, Zada YF, Dubé MP, Carleton BC, Hayden MR, CPNDS Consortium (2013). Replication of TPMT and ABCC3 genetic variants highly associated with cisplatin-induced hearing loss in children. Clin Pharmacol Ther.

[27] Xu H, Robinson GW, Huang J, Lim JY, Zhang H, Bass JK, Broniscer A, Chintagumpala M, Bartels U, Gururangan S, Hassall T, Fisher M, Cohn R (2015). Common variants in ACYP2 influence susceptibility to cisplatin-induced hearing loss. Nat Genet.

[28] Vos HI, Guchelaar HJ, Gelderblom H, de Bont ES, Kremer LC, Naber AM, Hakobjan MH, van der Graaf WT, Coenen MJ, te Loo DM (2016). Replication of a genetic variant in ACYP2 associated with cisplatin-induced hearing loss in patients with osteosarcoma. Pharmacogenet Genomics.

[29] Huang T, Cheng AG, Stupak H, Liu W, Kim A, Staecker H, Lefebvre PP, Malgrange B, Kopke R, Moonen G, Van De Water TR (2000). Oxidative stress-induced apoptosis of cochlear sensory cells: otoprotective strategies. Int J Dev Neurosci.

[30] el Barbary A, Altschuler RA, Schacht J (1993). Glutathione S-transferases in the organ of Corti of the rat: enzymatic activity, subunit composition and immunohistochemical localization. Hear Res.

[31] Galluzzi L, Senovilla L, Vitale I, Michels J, Martins I, Kepp O, Castedo M, Kroemer G (2012). Molecular mechanisms of cisplatin resistance. Oncogene.

[32] Ishimoto TM, Ali-Osman F (2002). Allelic variants of the human glutathione S-transferase P1 gene confer differential cytoprotection against anticancer agents in Escherichia coli. Pharmacogenetics.

[33] Gillet LC, Schärer OD (2006). Molecular mechanisms of mammalian global genome nucleotide excision repair. Chem Rev.

[34] Xiang T, Kang X, Gong Z, Bai W, Chen C, Zhang W (2017). XPG genetic polymorphisms and clinical outcome of patients with advanced non-small cell lung cancer under platinum-based treatment: a meta-analysis of 12 studies. Cancer Chemother Pharmacol.

[35] Rumiato E, Cavallin F, Boldrin E, Cagol M, Alfieri R, Basso D, Castoro C, Ancona E, Amadori A, Ruol A, Saggioro D (2013). ERCC1 C8092A (rs3212986) polymorphism as a predictive marker in esophageal cancer patients treated with cisplatin/5-FU-based neoadjuvant therapy. Pharmacogenet Genomics.

[36] Roco A, Cayún J, Contreras S, Stojanova J, Quiñones L (2014). Can pharmacogenetics explain efficacy and safety of cisplatin pharmacotherapy?. Front Genet.

[37] Xiao F, Pu J, Wen Q, Huang Q, Zhang Q, Huang B, Huang S, Lan A, Zhang Y, Li J, Zhao D, Shen J, Wu H (2017). Association between the ERCC2 Asp312Asn polymorphism and risk of cancer. Oncotarget.

[38] Lu J, Zhao H, Li S, Tian Z, Zhu X, Wang H, Fu H (2015). Correlation of rs1799793 polymorphism in ERCC2 and the clinical response to platinum-based chemotherapy in patients with triple negative breast cancer. Int J Clin Exp Med.

[39] Lambrechts S, Lambrechts D, Despierre E, Van Nieuwenhuysen E, Smeets D, Debruyne PR, Renard V, Vroman P, Luyten D, Neven P, Amant F, Leunen K, Vergote I (2015). Genetic variability in drug transport, metabolism or DNA repair affecting toxicity of chemotherapy in ovarian cancer. BMC Pharmacol Toxicol.

[40] Kanat O, Ertas H, Caner B (2017). Platinum-induced neurotoxicity: a review of possible mechanisms. World J Clin Oncol.

[41] Thiesen S, Yin P, Jorgensen AL, Zhang JE, Manzo V, McEvoy L, Barton C, Picton S, Bailey S, Brock P, Vyas H, Walker D, Makin G (2017). TPMT, COMT and ACYP2 genetic variants in paediatric cancer patients with cisplatin-induced ototoxicity. Pharmacogenet Genomics.

[42] Wheeler HE, Gamazon ER, Frisina RD, Perez-Cervantes C, El Charif O, Mapes B, Fossa SD, Feldman DR, Hamilton RJ, Vaughn DJ, Beard CJ, Fung C, Kollmannsberger C (2017). Variants inWFS1and Other Mendelian Deafness Genes Are Associated with Cisplatin-Associated Ototoxicity. Clin Cancer Res.

[43] Thomas JP (2006). High accumulation of platinum-DNA adducts in strial marginal cells of the cochlea is an early event in cisplatin but not carboplatin ototoxicity. Mol Pharmacol.

[44] Freyer DR, Chen L, Krailo MD, Knight K, Villaluna D, Bliss B, Pollock BH, Ramdas J, Lange B, Van Hoff D, VanSoelen ML, Wiernikowski J, Neuwelt EA (2017). Effects of sodium thiosulfate versus observation on development of cisplatin-induced hearing loss in children with cancer (ACCL0431): a multicentre, randomised, controlled, open-label, phase 3 trial. Lancet Oncol.

[45] Brock PR, Bellman SC, Yeomans EC, Pinkerton CR, Pritchard J (1991). Cisplatin ototoxicity in children: a practical grading system. Med Pediatr Oncol.

[46] Barahmani N, Carpentieri S, Li XN, Wang T, Cao Y, Howe L, Kilburn L, Chintagumpala M, Lau C, Okcu MF (2009). Glutathione S-transferase M1 and T1 polymorphisms may predict adverse effects after therapy in children with medulloblastoma. Neuro Oncol.

